# Accuracy of praxis test from Cambridge Cognitive Examination (CAMCOG) for Alzheimer’s disease: a cross-sectional study

**DOI:** 10.1590/1516-3180.2018.0022170418

**Published:** 2018-10-22

**Authors:** Juliana Francisca Cecato, Brian Alvarez Ribeiro de Melo, Gisele Correa de Moraes, José Eduardo Martinelli, José Maria Montiel

**Affiliations:** I MSc, PhD. Neuropsychologist and Professor, Instituto de Pós-graduação (IPOG) and Department of Internal Medicine, Faculdade de Medicina de Jundiaí (FMJ), Jundiaí (SP), Brazil.; II MSc, PhD. Statistician, Department of Statistics, Universidade Estadual de Maringá (UEM), Maringá, Paraná, Brazil.; III Student, Faculdade de Medicina de Jundiaí (FMJ), Jundiaí (SP), Brazil.; IV MD, PhD. Geriatrician and Professor, Department of Internal Medicine, Faculdade de Medicina de Jundiaí (FMJ), Jundiaí (SP), Brazil.; V MSc, PhD. Professor, Centro Universitário Fieo (UniFieo), Osasco (SP), Brazil.

**Keywords:** Dementia, Apraxias, Diagnosis, differential, Aged, Mental status and dementia tests

## Abstract

**BACKGROUND:**

Praxis impairment may be one of the first symptoms manifested in dementia, primarily in cortical dementia. The Cambridge Cognitive Examination (CAMCOG) evaluates praxis, but little is known about the accuracy of CAMCOG for diagnosing dementia. The aims here were to investigate the accuracy of praxis and its subitems in CAMCOG (constructive, ideomotor and ideational subitems) for diagnosing Alzheimer’s disease (AD) among elderly patients.

**DESIGN AND SETTING:**

Cross-sectional study on community-dwelling elderly people.

**METHODS:**

158 elderly patients were evaluated. CAMCOG, Mini-Mental State Examination and Pfeffer Functional Activities Questionnaire were used. ROC curve analysis was used to establish cutoff points.

**RESULTS:**

The total scores for praxis and the constructive subitem presented significant differences (P < 0.0001) between healthy elderly people and AD patients. Stage of dementia (clinical dementia rating, CDR = 0, 1 and 2) showed that total and constructive praxis can be used to classify the stages of dementia (mild and moderate cases), i.e. constructive praxis classified 88% of the patients with mild dementia (P < 0.0001) while total praxis classified 56% with moderate dementia. Comparison of normal controls (NC) and mild dementia cases showed specificity of 71% and sensitivity of 88% (AUC = 0.88; P < 0.0001).

**CONCLUSION:**

Some praxis subtests can have higher predictive diagnostic value for detecting Alzheimer’s disease in mild stages (total praxis AUC = 0.858; P < 0.0001; constructive AUC = 0.972; P < 0.0001). Constructive praxis as measured using CAMCOG may contribute towards diagnosing dementia, because occurrence of impairment of praxis may help in recognizing an evolving dementia syndrome.

## INTRODUCTION

The structured interview of the Cambridge Examination for Mental Disorders of the Elderly (CAMDEX)^1^ is widely used by Brazilian professionals and has been validated by Bottino et al. for the Portuguese language.^2^ Its cognitive tasks are called the Cambridge Cognitive Examination (CAMCOG) and evaluate several functions, such as memory, praxis, attention, orientation, perception, language and others.^1^ Aprahamian et al.^3^ and Nunes et al.^4^ (2008) found similar data regarding the accuracy of diagnostic investigation of dementia using CAMCOG. Sensitivity and specificity, respectively, were 100% and 95%. Even the reduced version of the cognitive battery, with only half of the items (CAMCOG-R), showed 98% sensitivity and 100% specificity for diagnosing Alzheimer’s disease (AD). In addition to use of CAMCOG for investigation of clinical conditions, Paradela et al.^5^ described its applicability in the context of epidemiological investigation. These authors pointed out the reliability of the total CAMCOG score: the patients were reassessed over a period of time and, even at different stages of dementia, the reliability of this score was maintained after analysis on internal consistency.

The relevance of praxis tasks as a form of reliable screening in relation to subcortical dementia such as major vascular neurocognitive disorder has been well documented in the literature.^6^^,^^7^^,^^8^^,^^9^ Evaluation of praxis in cases of subcortical dementia is important because this may demonstrate impairment in relation to execution of the tests; for example, through drawings (constructive task), gestures (ideational task) or a sequence of tasks (ideomotor task). This impairment is often inversely proportional to subcortical cerebral injury, thus allowing clinicians to provide a more accurate prognosis for cognitive decline and functional impairment.^8^^,^^9^^,^^10^^,^^11^^,^^12^

In 1920, Lipeman studied 84 patients who suffered strokes and discovered that, in addition to aphasia, the patients also had impairments of motor skills such as debilitated copying and imitative gestures.^13^ Moreover, regarding more precise aspects of the diagnosis, CAMCOG has contributed towards evaluation of total praxis and its subitems through showing the relevance of some studies that have indicated that impairment of praxis abilities confirms that there is a risk of rapid evolution to severe cases of dementia.^10^^,^^14^

## OBJECTIVE

The objective of the present study was to investigate the accuracy of the praxis test of CAMCOG for diagnosing Alzheimer’s disease among elderly people.

## METHODS

### Study design

This cross-sectional study was conducted in the city of Jundiaí, state of São Paulo. It was previously approved by the local institutional ethics committee (CEP number 853.742 and CAAEE number 34669514.0.000.5435) on January 1, 2015. All procedures were implemented in accordance with the Helsinki Declaration.

### Participants

The size of a representative sample was calculated as at least 101 participants (more details can be obtained in Fiel).^15^ Patients of both sexes, over 60 years of age, were evaluated. The initial sample comprised 237 elderly people, i.e. all the consecutive patients admitted between 2015 and 2017), who underwent anamnesis and neuropsychological evaluation. After the exclusion criteria had been applied, the study population comprised 158 participants. The following inclusion criteria were used: the participants needed to be men and women over 60 years of age, with one year of schooling or more; needed to have given their consent to voluntarily participate in the study; and needed to have signed the informed consent form.

In previous studies, the following exclusion criteria were adopted: presence of severe dementia (clinical dementia rating ≥ 3); history of stroke (according to magnetic resonance imaging examination); paralysis in both hands; depressive symptoms (scores ≥ 5 points on the Geriatric Depression Scale);^16^ walking using short steps; tremors and muscle rigidity that might suggest Parkinsonism; major tremors; visual and auditory difficulties; and neuropsychological reports of not being able to read and write (illiteracy).

AD participants were diagnosed with major neurocognitive disorder due to Alzheimer’s disease in accordance with DSM-5^17^ and NIA-AAW.^18^

### Praxis evaluation from CAMCOG

The CAMCOG cognitive battery was inserted as part of the CAMDEX investigation of mental disorders.^2^ CAMCOG has 67 cognitive items divided into subitems of memory, language, praxis, abstract thinking, calculus, attention, orientation, perception and gnosis.^1^^,^^2^ Application of the CAMCOG battery includes the Mini-Mental State Examination screening test.

CAMCOG evaluates three forms of praxis: constructive, ideational and ideomotor. In the constructive form of praxis, copies of figures such as a house in 3D and a pentagon are evaluated. In the ideomotor form of praxis, patients need to be able to perform learned tasks when receiving certain objects, for example, picking up a piece of paper and putting it inside an envelope. In the ideational form of praxis, patients need to be able to perform tasks in the correct order, such as making a “good-bye” movement with one hand or tying shoelaces.^19^^,^^20^

In this study, we used the CAMDEX structured interview subitems of the CAMCOG cognitive sections. These evaluate praxis by comparing the performance of elderly people with a diagnosis of AD with that of healthy elderly people (CG). The CAMCOG subitems for evaluating praxis were analyzed separately. These subitems were the following:

- Constructive praxis, in which the patient is asked to copy figures depicting a pentagon (1 point), a spiral (1 point), a house in 3D (1 point) and a clock (3 points). The total score for this subitem is 6 points;
- Ideational praxis, in which the patient is asked to place a paper inside an envelope (1 point);
- Ideomotor praxis, in which the patient is asked to follow the examiner’s commands. Three gestures are requested through these verbal commands, and the patient needs to be able to make the correct movements for them: a “goodbye” gesture; a movement of the fingers to indicate the action of cutting with a pair of scissors; and a gesture to show brushing the teeth (total score 5 points).

The total score possible for the praxis items was 12 points and a low score indicated impairment (apraxia).

### Data collection

All the cognitive tests were performed in a single session, lasting around 110 minutes. The diagnosis was determined after clinical, laboratory, neuroimaging and neuropsychological analyses. The patients underwent the Cambridge Cognitive Examination (CAMCOG),^1^^,^^2^ Mental State Mini-Examination (MMSE)^21^ (which is included in the CAMCOG battery), Geriatric Depression Scale questionnaire with 15 items^16^ and Pfeffer Functional Activities Questionnaire (PFAQ).^22^ It should be noted that the Geriatric Depression Scale was only applied as an exclusion criterion (depressive symptoms). The CAMCOG cognitive battery and the MMSE screening test were the instruments used to evaluate cognitive functions. The PFAQ was applied to obtain information about the patients’ performance in activities of daily life.

### Statistical methods

The data were analyzed using the Statistical Package for the Social Sciences (SPSS) software (version 15.0). Normality tests were performed, and occurrences of nonparametric distribution were indicated. Schooling, age and gender were analyzed in terms of percentages, means and standard deviations. To evaluate the effects of age, schooling and sex, correlations were made by controlling for these variables through Spearman analysis. Student’s test was performed for age and the chi-square test (χ^2^) was used between schooling and sex. For all analyses, the significance level was established as 5%.

Associative statistics between praxis and the memory subitems from CAMCOG were assessed. Correlations were made between praxis and diagnostic groups (CG and AD), separated according to schooling and age. As in Alzheimer’s disease, one of the earliest functions to be impaired is memory,^17^ and we chose to correlate this function from CAMCOG with praxis and its subitems. For this, we used the sum of the memory subitems from CAMCOG, i.e. the sum of the remote, recall, recent and recognition memories. A total memory score was generated, and it was this total score that was used for the correlation analysis.

Accuracy analyses were used to investigate the CAMCOG praxis at each of the three levels of states of dementia: no dementia, mild dementia and moderate dementia. These were measured using the Clinical Dementia Rating (CDR) scale.

Analyses between the diagnostic groups (AD and CG) in relation to the cognitive tests were made using the Mann-Whitney test. Finally, the sensitivity and specificity of the cognitive instruments (MMSE and CAMCOG) and praxis (total, constructive and ideomotor praxis) were analyzed by means of the receiver operating characteristic (ROC curve) and, for this analysis, the MedCalc software, version 15.8, was used.

## RESULTS

The 158 elderly subjects included in this study had a mean age of 78.62 years (minimum = 60, maximum = 97, standard deviation = 8.07) and 68.4% (n = 108) were female. Regarding schooling, 72.2% (n = 114) had had between 1 and 4 years, 12% (n = 19) between 5 and 8 years and 15% (n = 25) ≥ 9 years. 46.8% (n = 74) were diagnosed with Alzheimer’s disease (AD) and 53.2% (n = 84) formed the CG. Table 1 compares mean age, sex and schooling between the two groups. The sample was homogenous between the diagnostic groups (AD and CG) regarding the categorical variables (sex and education) and continuous variable (age).

**Table 1 .** Characteristics among diagnostic groups in relation to age, sex and schooling. Descriptive data from MMSE, CAMCOG, praxis (total score and sub-items) and PFAQ on 74 patients diagnosed with Alzheimer’s disease (AD) and 84 healthy elderly people (control group, CG)

	CG	AD	P
Age (years)	77.73 (60-96) (± 8.66)	79.64 (60-97) (± 7.28)	*0.139
Sex			
Female	66.7%	70.3%	**0.628
Male	33.3%	29.7%	
Schooling			
1 to 4 years	71.4%	73%	**0.730
5 to 8 years	10.7%	13.5%	
> 9 years	17.9%	13.5%	
	Mean ± SD	Mean ± SD	
MMSE	27.45 ± 2.43	17.05 ± 4.25	0.0001
CAMCOG	89.9 ± 9.39	56.35 ± 13.84	0.0001
PFAQ	0.77 ± 1.97	19 ± 9.10	0.0001
Praxis (total)	10.82 ± 1.32	8.30 ± 1.86	0.0001
Constructive	5.63 ± 0.70	3.36 ± 1.54	0.0001
Ideational	0.95 ± 0.21	0.95 ± 0.22	0.854
Ideomotor	4.21 ± 0.89	3.99 ± 0.97	0.114

MMSE = Mini-Mental State Examination; CAMCOG = Cambridge Cognitive Examination; PFAQ = Pfeffer Functional Activities Questionnaire; SD = standard deviation. *P from Student t test; **P from chi-square test.

We investigated the sensitivity and specificity data relating to the CAMCOG praxis item and its sub-items, from both diagnostic groups: CG and AD. Table 1 describes the comparison between the two diagnostic groups in relation to the cognitive tests (MMSE, CAMCOG and praxis) and the subitems (constructive, ideational and ideomotor). There were lower means in the AD group in all cognitive tests except for the ideational and ideomotor subitems. It can be inferred that the MMSE (P < 0.0001), CAMCOG (P < 0.0001), total praxis (P < 0.0001) and constructive praxis (P < 0.0001) tests were able to statistically differentiate between the two diagnostic groups. Lower mean scores were observed in the group with the diagnosis of AD. It is important to note that the group with AD scored below the cutoff point for CAMCOG, which would be above 80 points. CAMCOG is considered to be the diagnostic tool for mental disorder among elderly people.^1^^,^^2^ The statistical differences that were found between MMSE and CAMCOG were concordant with data in studies in the literature,^3^^,^^4^^,^^23^ which emphasizes the high sensitivity and specificity of these instruments (MMSE and CAMCOG) for diagnostic investigation of AD.

The ideational (P = 0.854) and ideomotor (P = 0.114) subitems were not able to differentiate between the two groups. It can be inferred that both of these subitems have a ceiling effect, i.e. they provide satisfactory scores in both the healthy elderly group and the group with neurocognitive disorder (Table 1). Although Nagahamaet et al.^8^ and Trojano et al.^9^ showed statistically significant differences regarding the ideational and ideomotor subitems for identifying subcortical impairment, our findings indicate that in cases of predominantly cortical dementia, these subitems are preserved even in cases of evident disorder.

The ideational subitem consists of only one score (1 point for a correct result and 0 point for an erroneous result). On the other hand, the ideomotor subitem presents a maximum score of 5 points, but this task can be satisfactorily performed by patients with mild and moderate dementia because of the automation of the act (i.e. it can be done even with a lack of comprehension).^24^^,^^25^ However, there were statistically significant differences in relation to the items of total praxis (P < 0.0001) and constructive praxis (P < 0.0001).

There was no correlation between the variables and the subitems of praxis. Only constructive praxis presented a tendency to be associated with the educational variable (*r* = 0.23; P = 0.052). As in Alzheimer’s disease, one of the earliest functions to be impaired is memory, and we chose to correlate this function from CAMCOG with praxis and its subitems. Robust positive correlation coefficients between memory and total praxis (*r* = 0.72; P < 0.0001) and between memory and constructive praxis (*r* = 0.71; P < 0.0001) could be seen. A weak positive correlation between memory and ideomotor praxis (*r* = 0.34; P < 0.0001) was found. There was no correlation between memory and ideational praxis (*r* = 0.17; P = 0.077).

Table 2 shows that ideomotor praxis cannot differentiate between mild and moderate dementia. Moreover, it can be seen that total and constructive praxis can be used to classify the stages of dementia (mild and moderate cases), i.e. constructive praxis classified 88% of the patients with mild dementia while total praxis classified 56% with moderate dementia (P < 0.0001). Comparing the control group (NC) and moderate dementia group (CDR = 2), it can be seen that constructive praxis correctly classified 96% of the patients in the control group and 81% of those with moderate dementia (CDR = 2). Comparison of the control group and mild dementia group showed specificity of 71% and sensitivity of 88% (P < 0.0001), as presented in Table 2.

**Table 2.** Accuracy of praxis instruments for levels of clinical dementia rating (CDR). CDR = 0, no dementia; CDR = 1, mild; CDR = 2, moderate

CDR	Instruments	AUC	P	95% CI	Sensitivity	Specificity	Cutoff point
0-1	Total praxis	0.82	0.0001	0.742-0.900	0.64	0.85	9
	Constructive praxis	0.88	0.00	0.808-0.946	0.88	0.71	5
	Ideomotor praxis	0.56	0.29	0.453-0.665	0.69	0.44	4
0-2	Total praxis	0.92	0.0001	0.854-0.980	0.78	0.94	8
	Constructive praxis	0.93	0.0001	0.875-0.995	0.81	0.96	3
	Ideomotor praxis	0.57	0.21	0.452-0.697	0.31	0.85	3
1-2	Total praxis	0.74	0.0001	0.623-0.856	0.59	0.79	7
	Constructive praxis	0.74	0.0001	0.624-0.863	0.56	0.88	2
	Ideomotor praxis	0.53	0.69	0.389-0.667	0.19	0.98	2

AUC = area under the curve; CI = confidence interval.

**Figure 1.**
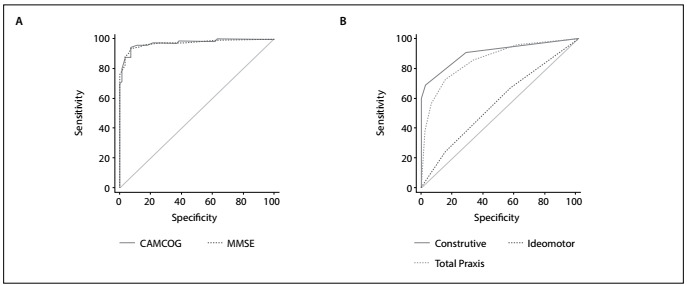
Graphical analysis on receiver operating characteristic (ROC) curve in relation to Cambridge Cognitive Examination (CAMCOG), Mini-Mental State Examination (MMSE), praxis and subitems. (A) Comparison of area under the curve (AUC) between MMSE and CAMCOG instruments. (B) Comparison of AUC among total praxis, constructive subitem and ideomotor subitem. Graph (B) shows that constructive praxis presents the largest value.

We had the objective of comparing the sensitivity and specificity data between MMSE and CAMCOG. The analyses performed using the CAMCOG cognitive battery showed 95% sensitivity and 93% specificity, and the cutoff point observed for this sample was 75 points (Table 3 and Figure 1). The MMSE presented sensitivity of 93% and specificity of 93%, and the cutoff point was 23 points (Table 3 and Figure 1).

**Table 3.** Accuracy of different tests for diagnosing Alzheimer’s disease

Instruments	AUC	P	95% CI	Sensitivity (%)	Specificity (%)	Cutoff point
CAMCOG	0.975	< 0.0001	94-99	95	93	75 points
MMSE	0.972	< 0.0001	93-99	93	93	23 points
Constructive praxis	0.905	< 0.0001	85-95	69	98	4 points
Total praxis	0.858	< 0.0001	79-90	73	84	9 points
Ideomotor praxis	0.568	0.111	49-65	66	44	4 points

AUC = area under the curve; CI = confidence interval; CAMCOG = Cambridge Cognitive Examination; MMSE = Mini-Mental State Examination. P from chi-square test.

Analyses on the ROC curve were performed only for the praxis subitems and the total praxis item (12 points). Only ideational praxis, which received 1 point for correct execution, was not assessed through the ROC curve. In relation to the constructive subitem, the cutoff point was taken to be 4 points, which had sensitivity of 69% and specificity of 98% (Table 3 and Figure 1). ROC curve analysis on the ideomotor subitem presented sensitivity of 66% and specificity of 44% for a cutoff point of 4 points (Table 3 and Figure 1), i.e. lower values than those of the constructive subitem. Finally, the ROC curve methodology was performed for total praxis through CAMCOG, in order to verify this ability (by adding the scores for the three subitems: constructive, ideomotor and ideational). Table 3 and Figure 1 show that this presented sensitivity of 73% and specificity of 84%, with a cutoff point of 9 points.

Given that the aim of this study was to investigate sensitivity data relating to praxis and its subitems through CAMCOG, the findings demonstrated that there was a decline in Alzheimer’s disease patients, particularly regarding constructive praxis.

## DISCUSSION

The aim of this study was to investigate the sensitivity of praxis data from CAMCOG for evaluations on elderly people with major neurocognitive disorders. Our results showed that the mean scores for total praxis and its constructive subitem were higher among healthy elderly people, with a statistically significant difference, as shown in Table 1. This result can be explained by the structural brain alterations that occur in AD patients (cortical dementia with temporoparietal impairment). Involvement of the motor cortex (parietal lobe) was responsible for the alterations that were found in this evaluation. It has been reported that there is a risk that impairment of praxis skills will rapidly evolve in cases of dementia.^10^^,^^19^ One hypothesis explaining the aggressive evolution of dementia in patients who present early impairment of praxis is that this may be related to degeneration of the temporal and parietal areas, i.e. the regions involved in the circuits for praxis. This hypothesis was developed through the observation that some patients evolve more slowly, while others evolve significantly faster.^13^

The statistically significant difference relating to constructive praxis may be explained by the theory that many brain regions in both hemispheres are involved in different aspects of the design copy test. This could provide an explanation for the findings of this study, through the suggestion that declines in constructive praxis are related to impairment of cognitive abilities in cases of cortical dementia, such as Alzheimer’s disease.^9^^,^^26^^,^^27^ The hypothesis raised from this finding is that constructive praxis provides an index for cognitive deterioration. This means that as AD progresses, it will compromise both the left and the right hemisphere diffusely.^26^^,^^27^^,^^28^

Analysis of praxis abilities is important in examining motor behavior, in terms of activities of daily life. The data from our study showed that praxis declined (for both total score and the constructive subitem). This may suggest that, even at mild stages of dementia, it is problematic for elderly people to continue to drive (Table 2). Patients at moderate stages of dementia present significant inability to deal with tasks such as driving or cooking. Driving depends on motor skills, such as praxis abilities. Driving also depends on attention, working memory and processing speed. Although only one of the skills may be impaired, management of elderly patients with major neurocognitive disorder becomes both a problem for the family and a public health problem, in that elderly drivers should be evaluated.^29^ There is a need to review the praxis items within CAMCOG, so as to be able to assess the skills of elderly drivers. Impaired driving skills are only one example of the negative debilitating effects of apraxia, but it is important to emphasize the general importance of evaluation of praxis, as one of the indicators of cognitive fragility among elderly patients.^30^^,^^31^ Our results, presented in Table 2, correlated the stages of dementia (CDR) with the scores for total praxis and its constructive and ideomotor subitems. This may suggest that these items are related to stages of dementia.

Regarding constructive praxis, there is a requirement for visual skills and motor planning. Both cerebral hemispheres act towards accomplishment of constructive tasks. Errors are usually associated with right-hemisphere parietal lesions due to deficits of perception, while errors of execution are related to lesions in the left hemisphere. Ideomotor apraxia is related to lesions in the parietal cortex of the left hemisphere, in the corpus callosum and in the basal ganglia. Ideational apraxia is usually caused by severe disturbances in the temporal sequence of motor actions.^11^ Assessment of constructional praxis has been extensively used in diagnostic investigations of dementia syndrome. Some tests such as the clock drawing test (CDT) and copying of pentagons are considered to be more complex because they involve organization and planning of the motor action in order to carry out the task and are also influenced by schooling level.^14^ Those findings corroborate the data of the present study, which found a greater area under the curve (AUC) in the constructional subitem (AUC = 0.905) than in the total praxis item (AUC = 0.858) and the ideomotor subitem (AUC = 0.568). This explains the weak correlation between memory and praxis, since the same brain regions may not be involved in the same satisfactory performances.

Chandra et al.^13^ reported that this cognitive function (ideomotor, constructive and ideational praxis) was important in relation to corticobasal degeneration (encompassing cerebral cortex and basal ganglia). In other words, from the time when neuronal loss occurs in the cortical region and basal ganglia (participating in motor circuits), patients will present impairment of intentional execution of motor tasks. There may be impairment in the early stages of dementia syndrome. Motor areas of the cortex send signals to the basal ganglia and these in turn replicate the motor signals that are transmitted to the parietal cortex. Any failure to communicate or send signals can cause apraxia.

While memory disorders tend to dominate cognitive psychology and neuropsychology, praxis deficits have been placed in the background. This often leads to difficulties in accurately interpreting the nature of motor disorders presented by patients with neurological and neuropsychiatric disorders.^24^ Johnen et al.^30^ and Hazan et al.^14^ described these concerns in relation to aging populations and indicated that there is a need for physicians and other healthcare professionals to have access to screening tools with predictive value for identifying cognitive impairment in cases of suspected dementia. These authors stated that such instruments would need to have high diagnostic accuracy and be fast and easily administered, and that praxis tests might be able to fulfill this purpose.

Our findings demonstrated that, as screening tests, MMSE had high diagnostic efficacy (AUC = 0.972), while CAMCOG presented a satisfactory AUC = 0.975 value. Helmes^19^ and Martinelli et al.^32^ critically appraised the pentagon drawing test that forms part of MMSE and stated that this is an important test that evaluates organic brain dysfunction, even though it only receives a dichotomous score. These authors also stated that this test of copying a pentagon is so important for evaluating cerebral dysfunction that it should be scored independently. Our results have made us think about using the praxis item as a screening tool, because the constructive subitem (AUC = 0.905; sensitivity = 69%; specificity = 98%) was more effective in investigating cognitive impairment than was the total praxis item (AUC = 0.858; sensitivity = 73%; specificity = 84%). These findings agree with those of the studies by Hazan et al.,^14^ Helmes,^19^ Johnen et al.^30^ and Martinelli et al.,^32^ thus indicating that the most effective praxis screening tests are those that comprise constructive tasks.

Evaluation of apraxia among elderly people is a way of exploring the field of cognition as part of the diagnostic investigation of neurodegenerative diseases. It was found through the praxis subitems from CAMCOG that some patients, even those whose aging process is healthy, present some difficulties in performing such functions with accuracy.

One limitation of the present study was in relation to the severity of dementia. Moreover, we only evaluated the forms of praxis that are included in CAMCOG, which only considers three of the various types of apraxia, such as dynamic, myokinetic, gait, dressing, buccofacial, agnostic and diagnostic apraxia.

## CONCLUSION

Some praxis subtests may have higher predictive diagnostic value in detecting Alzheimer’s disease in mild stages. However, only constructive praxis from CAMCOG showed higher accuracy for identifying dementia. Our contribution from the present study consists of the suggestion that cognitive screening tasks consisting of constructive praxis should be used (i.e. copying of figures) and that this cognitive information could be particularly appropriate for investigating impairment in patients with suspected dementia. In addition to use of constructive praxis in screening tests, we also found that it was effective in screening for Alzheimer’s disease and thus was an effective test for predominantly cortical dementia.
